# Complete Genome Sequence of the Thermophilic Enterococcus faecalis Strain K-4, Isolated from a Grass Silage in Thailand

**DOI:** 10.1128/mra.00814-22

**Published:** 2023-03-27

**Authors:** Meimi Kuwabara, Rei Irimajiri, Shun Togo, Yasuhiro Fujino, Masanori Honsho, Shiro Mawatari, Takehiko Fujino, Katsumi Doi

**Affiliations:** a Microbial Genetic Division, Institute of Genetic Resources, Faculty of Agriculture, Kyushu University, Fukuoka, Japan; b Department of Neuroinflammation and Brain Fatigue Science, Graduate School of Medical Sciences, Kyushu University, Fukuoka, Japan; c Institute of Rheological Functions of Food, Fukuoka, Japan; University of Maryland School of Medicine

## Abstract

The whole-genome sequence of strain K-4, isolated from grass silage in Thailand, which constitutes a chromosome and two plasmids, is 2,914,933 bp long, has a GC content of 37.5%, and contains 2,734 predicted protein-coding genes. Average nucleotide identity based on BLAST+ (ANIb) and digital DNA-DNA hybridization (dDDH) values indicated that the strain K-4 was closely related to Enterococcus faecalis.

## ANNOUNCEMENT

The genus *Enterococcus* comprises Gram-positive, non-spore-forming facultative anaerobic, spherical, or ovoid cells with low genomic GC content (1, 2). Based on 16S rRNA genome sequencing, *Enterococcus* members can be divided into Enterococcus faecalis, Enterococcus avium, Enterococcus faecium, Enterococcus cecorum, Enterococcus gallinarum, and Enterococcus italicus groups (3). Some *Enterococcus* strains have been reported to produce antimicrobial compounds, such as bacteriocins (4). E. faecalis strain K-4 produces enterocin SE-K4, which is carried on plasmid pEK4S (5). Strain K-4 is thermostable, and enterocin SE-K4 belongs to the leucocin A/sakacin P family (6). Although over 6,000 E. faecalis genomes have been deposited, few bacteriocin-producing strains have been characterized.

Strain K-4 was isolated from a grass silage in Bangkok, Thailand (6, 7), and was stored as glycerol stock in a deep freezer (−80°C) at our institute until use. It was revived and cultivated in M17 medium (BD Difco, USA) at 30°C overnight; thereafter, genomic DNA for both long- and short-read sequencing was isolated using Marmur’s method (8).

Short-read sequencing libraries were constructed using the NEBNext Ultra II FS DNA library prep kit (New England BioLabs [NEB], USA) and decoded on the Illumina MiSeq system. Overall, 1,192,876 reads with 299,930,918 bases were decoded with an average read length of 251.4 bp and spot length of 602 bp with 2 × 301-bp paired-ends reads. Low-quality bases (Q scores, <15) were trimmed, and short reads (<25 bp) were removed using Platanus trim v1.0.7 (http://platanus.bio.titech.ac.jp/pltanus_trim) (9). Long-read sequence libraries were constructed using the rapid barcoding kit (SQK-RBK004), and sequencing was performed on a MinION device (Oxford Nanopore Technologies [ONT], UK) using an R9.4.1 flow cell. No size selection was performed before library preparation. Overall, 111,821 reads with 580,338,872 bases were decoded using Guppy v4.0.15 (10). Data recovered with NanoFilt v2.7.1 (11) (quality, ≥10; length, ≥2 kb; head crop, 100 bp) revealed a mean read length of 8,717.7 bp and *N*_50_ value of 12,332 bp. Three contigs that resulted from long- and short-read sequencing (chromosome and two plasmids) were assembled *de novo* using Unicycler v0.4.8 (https://github.com/rrwick/Unicycler) (12), which was also used to rotate the single contig to start with *dnaA* for the chromosome. The genome sequence was annotated with DFAST v1.2.6 (https://dfast.nig.ac.jp/) (13). Average nucleotide identity based on BLAST+ (ANIb) and digital DNA-DNA hybridization (dDDH) values were calculated via the JSpeciesWS online service v3.9.0 (http://jspecies.ribohost.com/jspeciesws/#analyse) and the Genome-to-Genome Distance Calculator 3.0 (http://ggdc.dsmz.de/ggdc.php) (14) using reported genome sequences of type strains of the E. faecalis group. Default parameters were used for all software unless otherwise specified.

One contig of the assembled genome sequence was 2,790,927 bp (GC content of 37.5%) with a sequence depth of ~88. Overall, 2,734 coding regions, 60 tRNAs, 12 rRNAs, and 1 CRISPR region were annotated. The DNA sequences of two plasmids, namely, pEK4S (59,411 bp) and pEK4L (64,595 bp), were also assembled and compared with their partial sequences using Blast (5, 6). Based on ANIb and dDDH results, strain K-4 was 97.7% and 90.1% similar, respectively, to E. faecalis ATCC 19433^T^ (Table 1).

**TABLE 1:** Average nucleotide identity and digital DNA–DNA hybridization values for Enterococcus faecalis strain K-4 compared with other *Enterococcus* strains

Species	Comparison strain		ANIb (%)	dDDH (%)
	Name	GenBank accession no.		
Enterococcus faecalis	E. faecalis 76EA1	NZ_KZ846109.1	99.8	99.3
	E. faecalis NCTC 8131	NZ_UGIU01000002.1	99.8	99.1
	E. faecalis ATCC 19433^T^	KB944590.1	97.7	90.1
	Enterococcus caccae ATCC BAA-1240^T^	NZ_KE136472.1	72.2	20.2
	Enterococcus haemoperoxidus ATCC BAA-382^T^	NZ_KE136479.1	73.0	21.5
	Enterococcus moraviensis ATCC BAA-383^T^	NZ_KB946318.1	72.2	19.9
	Enterococcus silesiacus LMG 23085^T^	CP013614.1	73.2	21.8
	Enterococcus termitis LMG 8895^T^	NZ_MIJY01000001.1	72.1	20.2
Enterococcus avium	*E. avium* ATCC 14025^T^	NZ_KE136357.1	66.2	28.9
Enterococcus cecorum	*E. cecorum* NCTC 12421^T^	LS483306.1	70.6	26.1
Enterococcus faecium	E. faecium NBRC 100486^T^	NZ_BCQD01000001.1	71.0	23.5
Enterococcus gallinarum	*E. gallinarum* NCTC 12359^T^	NZ_UFYU01000004.1	70.7	30.8
Enterococcus italicus	*E. italicus* DSM 15952^T^	GL622241.1	69.8	21.6

### Data availability.

Strain K-4 genome sequence and annotation data were deposited in DDBJ/GenBank under BioProject number PRJDB12421; BioSample number SAMD00412537; DRA numbers DRA012938 and DRA012951; accession numbers AP025270, AP025271, and AP025272; and SRA numbers DRX313729 (MinION) and DRX314299 (Illumina).
